# Measuring Implicit Approach‐Avoidance Tendencies Using Self‐Depicting Body Pictures in Female Adults With Bulimia Nervosa, High Body Dissatisfaction and Healthy Controls

**DOI:** 10.1002/eat.24523

**Published:** 2025-08-09

**Authors:** Johanna Xemaire, Ines Wolz, Dustin Werle, Carolin Dudschig, Jennifer Svaldi

**Affiliations:** ^1^ Department Clinical Psychology and Psychotherapy University of Tübingen Tübingen Germany; ^2^ Department Language and Cognition University of Tübingen Tübingen Germany

**Keywords:** approach avoidance task, body dissatisfaction, body‐avoidance, bulimia nervosa

## Abstract

**Objective:**

Body dissatisfaction is an important factor for the etiology and maintenance of eating disorders such as bulimia nervosa (BN). At the behavioral level, body dissatisfaction often manifests itself in excessive body‐related avoidance, thought to act as a negative reinforcer. The Approach‐Avoidance Task (AAT) is an implicit measure of avoidance behavior, but the literature on body‐related avoidance measured by the AAT is inconclusive. In the present study, we considered self‐reference and cognitive load as important dimensions to better understand AAT‐evoked biases.

**Method:**

Adult female participants with BN (*n* = 21), high body dissatisfaction (*n* = 20), and healthy controls (*n* = 20) completed a novel, slider‐based AAT with task‐irrelevant self‐depicting body pictures and scrambled versions of these pictures as control stimuli. We further induced cognitive load through a flanker task to assess possible moderating effects.

**Results:**

There was no significant Group × Picture Type × Motion Direction interaction for either motion onset or motion duration. The results further revealed a standard flanker effect in that participants reacted faster to compatible trials; but this was independent of Group membership, Picture Type, and Motion Direction.

**Discussion:**

In sum, the AAT did not yield evidence of body‐related avoidance behavior. Future studies should control for the occurrence of body checking (i.e., increased focus on disliked body parts which could activate approach‐biases) during AAT, manipulate the strength of self‐reference, e.g., by presenting/omitting facial cues in self‐depicting body pictures, and consider the task relevance of (and thus overt attention to) the body pictures.

**Summary**

- Own‐body avoidance is thought to be a key factor in maintaining body dissatisfaction and thus eating disorders.
- However, in this study, females with bulimia nervosa and high body dissatisfaction did not show implicit body avoidance when confronted with pictures of their own body.
- This null finding and its contextualization inform future research to understand the relationship between implicit and explicit measures of body avoidance.

## 1 Introduction

Body dissatisfaction (BD) is highly prevalent in western societies and an empirically validated risk factor for the development (Fallon et al. 2014; Fiske et al. 2014; Jaeger et al. 2001; Stice 2016) and maintenance of eating disorders (Dakanalis et al. 2017; Stice 2002). A negative body image in individuals with eating disorders is further associated with a poorer treatment response (Fichter et al. 2008; Hilbert et al. 2007; Masheb and Grilo 2008; Wilson et al. 1999) and higher relapse rates (Freeman et al. 1985; Keel et al. 2005). A better understanding of the mechanisms involved in the maintenance of BD seems, therefore, mandatory to improve current gold‐standard prevention and treatment approaches for body image disturbances.

From a theoretical perspective, both cognitive and behavioral factors are thought to maintain BD (Williamson et al. 2004). Within the cognitive domain, cognitive biases pertaining to attention, memory, and interpretation of body‐related stimuli (Paslakis et al. 2021; Rodgers and DuBois 2016) and dysfunctional use of emotion regulation strategies after activation of body‐related schemata (Leppanen et al. 2022; Naumann et al. 2016) have been shown to differentiate individuals with high versus low BD. Additionally, behavioral factors such as body‐checking and body‐related avoidance are also thought to play a key role in this vicious cycle.

Research into body image exposure further indicates that, when confronted with their body, individuals with bulimia nervosa (BN) and with high BD exhibit heightened stress responses, as evidenced by increased arousal, negative affect, and autonomic response (Baker et al. 1995; Baur et al. 2020; Coelho et al. 2013; Tuschen‐Caffier et al. 2003). Therefore, they often engage in body avoidance, such as covering mirrors and wearing oversized clothing. From a theoretical perspective (Williamson et al. 2004), these behaviors are believed to provide temporary relief. Ultimately, however, these behaviors impede the disconfirmation of dysfunctional body‐related cognitions, thereby reinforcing BD (Nikodijevic et al. 2018; Walker et al. 2018).

So far, several studies reported body‐related avoidance by subjective or by more implicit measures such as physiological responses during body exposure. Beyond this, body‐related avoidance may also be evident in implicit behavioral reactions towards aversive stimuli (Woud et al. 2011). In contrast to self‐report, implicit measures are less susceptible to response biases such as social desirability (Fisher 1993). Therefore, expanding the research to implicit measures of avoidance is paramount to a comprehensive understanding of the mechanisms underlying BD.

The Approach Avoidance Task (AAT; Heuer et al. 2007; Rinck and Becker 2007) enables the measurement of automatic avoidance tendencies. The AAT is grounded on the assumption that humans tend to approach pleasant and avoid unpleasant stimuli. Notably, there are several different variants of this task. In the two most established, participants either move the target stimulus towards or away from themselves by operating a joystick, or they move a manikin figure representing themselves away or towards the stimulus. The theoretical principle underlying the task is that when using a joystick, approach behavior is associated with arm flexion, i.e., pulling something towards oneself, while avoidance behavior is associated with arm extension, i.e., pushing something away.

These behaviors are often further visualized by zooming in or out on the stimulus image to create a 3‐D effect. When participants are asked to pull neutral/pleasant pictures (or move the manikin towards) and push (or move the manikin away from) aversive stimuli, this is considered a compatible response; whereas pulling (moving the manikin towards) aversive pictures and pushing (moving the manikin away from) pleasant pictures would be incompatible. Many studies have shown that, relative to compatible trials, incompatible trials lead to slower reaction times and correlate with subjective aversion regarding the picture content (e.g., Heuer et al. 2007; Klein et al. 2011; Loijen et al. 2020; Mogg et al. 2005; Rinck and Becker 2007; Zech et al. 2020).

Notably, research employing the AAT to investigate implicit behavioral approach‐avoidance tendencies toward body stimuli in individuals with high BD and/or eating disorders is scarce and—at least in regard to the processing of bodies other than the self‐body—inconclusive (for an overview on studies using different implicit measures to investigate body biases see Paslakis et al. (2021)).

As such, one study found an approach bias towards thin bodies in female students. However, in this study, there was no evidence for an avoidance of the same body images stretched 20% representing “chubby” bodies (Woud et al. 2011). Two further AAT studies including females with and without self‐reported eating disorder symptoms as well as participants diagnosed with BN (Kollei et al. 2022; Leins et al. 2018) suggest a general avoidance bias in regard to (computer generated) body images irrespective of group membership. Furthermore, this avoidance bias was independent of the weight status of the depicted stimuli.

Surprisingly, in another AAT study that used more naturalistic stimuli (instead of artificially created “chubby” ones in the Woud et al. 2011 study and computer‐generated bodies in the Kollei et al. 2022 and Leins et al. 2018 study), female students with subclinical levels of disordered eating displayed a relatively faster approach to thin bodies and a relatively faster avoidance of overweight bodies (Dondzilo et al. 2019). Additionally, the approach bias in this study was significantly positively correlated with greater self‐reported BD, thin‐ideal internalization, and dietary restraint. However, in a similar population, a follow‐up study using black and white images of under‐ and overweight bodies found opposite results in that participants displayed a general avoidance of underweight bodies but a tendency to approach overweight bodies (Dondzilo et al. 2021). Furthermore, in this study, the approach bias towards overweight bodies was significantly and positively correlated with eating disorder pathology. In contrast to the other studies, this latter study used a more ecologically valid touchscreen‐based task, in which approach and avoidance behaviors had to be executed by arm movements (rather than joystick applications) simulating better spatial distance.

Several aspects have been discussed to account for the heterogeneity of results. First of all, some studies assessed approach‐avoidance tendencies by joystick applications (Kollei et al. 2022; Leins et al. 2018); others by touchscreen involving arm movements (rather than hand movements only) (Dondzilo et al. 2021), and some studies used manikin‐based tasks (Dondzilo et al. 2019; Woud et al. 2011). Second, studies differ in regard to the task‐relevance of the presented stimuli. As such, some studies instructed their participants to respond to the perceived weight of the body stimuli (Dondzilo et al. 2019; Dondzilo et al. 2021; Woud et al. 2011), whereas in other studies (Kollei et al. 2022; Leins et al. 2018) the physical appearance of the body stimuli was irrelevant to the task and participants were instructed to respond to the color of the underwear. At a conceptual level, task‐irrelevant approaches may tackle more implicit associations (De Houwer and Moors 2007), while task‐relevant approaches may tap more explicit associations. Notably, the evidence regarding coherence between more implicit and more explicit measures is mixed; some studies found no coherence at all (Gawronski et al. 2020).

Stimulus properties may play an additional role for the AAT to produce reliable approach‐avoidance patterns across diagnostic groups. For example, studies differ widely in regard to the type of body stimuli presented. While some employed computer‐generated body stimuli (Kollei et al. 2022; Leins et al. 2018), others utilized photographs of actual bodies, albeit with modifications such as image stretching (Woud et al. 2011); in some studies, images were cropped to only display specific body parts (Dondzilo et al. 2019), while others presented them in grayscale (Dondzilo et al. 2021).

Of relevance, cognitive theories of eating disorders emphasize that body dissatisfaction emerges from negative body‐related *self*‐schema (e.g., Williamson et al. 2004). Indeed, in one study (Brockmeyer et al. 2020), the presence of body‐related approach–avoidance bias was mediated by self‐reference. In particular, the study revealed that females with anorexia nervosa (AN) exhibited an approach bias towards thin bodies, solely when the participants' faces were superimposed over these images. In the healthy control group, no such difference was found. Thus, not only do action tendencies to body cues depend on the stimulus material and the ecological validity of the task, but also self‐reference seems to be particularly relevant, possibly by increasing attentional focus (Tacikowski and Nowicka 2010). Finally, to the best of our knowledge, only one study to date has used self‐depicting (own) body images to investigate action tendencies towards body cues in participants with AN, BN, and healthy controls (Legenbauer et al. 2020) and found no significant differences in approach tendencies in dependence on group membership. However, the study used a body image *approach* task; therefore, no conclusions can be drawn about avoidance tendencies.

The present study aimed to advance the field of AAT research regarding body‐avoidance by introducing a novel, slider‐based AAT to investigate approach and avoidance behaviors using self‐depicting body images in a group of females with BN, a group of females with high BD without BN (BD^+^), and a control group (HC) of females without BN scoring low on BD. We used diffeomorphically scrambled versions of the self‐body images (i.e., images where the original pixels were systematically warped using a mathematical algorithm based on diffeomorphism) as control stimuli (Press et al. 2022). These diffeomorphic stimuli have the advantage of being no longer recognizable as body images while still preserving the basic lower‐level visual stimulus properties of the self‐body images from which they originated (e.g., perceptual organization, spatial frequency).

We hypothesized a self‐body avoidance bias (i.e., faster responses on trials in which participants are instructed to push self‐body stimuli away from themselves relative to trials in which body stimuli must be pulled towards the participant) in the BN and the BD^+^ group; whereas no such bias was expected for the HC group. No approach‐avoidance bias across groups was expected for neutral images.

Notably, in several previous implementations of the AAT, the stimuli used to assess approach‐avoidance tendencies were task relevant; that is, participants had been instructed to either pull the body‐related stimuli towards themselves or to push them away from themselves (Dondzilo et al. 2019; Dondzilo et al. 2021; Woud et al. 2011). In the present study, we employed a flanker task to render the body images irrelevant to the task. Commonly, flankers are embedded in proximity to the task stimuli. In the present study, however, the flankers were superimposed on the body‐related stimuli to ensure that participants would not divert their attention away from the stimuli to perform the task. Using task‐irrelevant body images offers the advantage of measuring more automatic associations (De Houwer and Moors 2007), while also maintaining consistency in participants' instructions throughout the experiment and thus avoiding order effects when modifying the cognitive load during AAT trials. Given that some studies suggest that cognitive load increases disinhibition and impulsivity (Boon et al. 2002; Vasquez 2009; Ward and Mann 2000) as well as avoidance tendencies (Cezar and Maçada 2023), while other studies have found the opposite effect, with cognitive load decreasing disinhibition and impulsivity (Sharbanee et al. 2014; Van Dillen et al. 2013), we wanted to assess whether high versus low cognitive load (incompatible vs. compatible flanker tasks, respectively) affects the hypothesized avoidance bias (exploratory analysis).

## 2 Method

### 2.1 Sample

We recruited a total sample size of 61 females (assigned female sex at birth) for a power of 0.80 to detect a 50 ms difference between push and pull responses for self‐body images in the BN and BD^+^ groups as specified in our power simulation (see Appendix S1). Exclusion criteria for the HC were a lifetime diagnosis of any eating disorder and a score on the body shape questionnaire (BSQ) of > 67, which refers to the lowest three deciles in reference to a community sample (Cooper et al. 1987). Participants in the BD^+^ group were required to have values ≥ 96 on the BSQ, which refers to the top three deciles in a community sample (Cooper et al. 1987). Inclusion into the BN group was based on the DSM‐5 criteria (American Psychiatric Association 2013). A lifetime diagnosis of any eating disorder was not defined as an exclusion criterion for either the BD^+^ group or the BN group. However, participants could only be included in the BD^+^ group if they did not meet the diagnostic criteria for any current eating disorder, including subclinical and other specified eating disorders. For further information on exclusion criteria and sample recruitment, see Appendix S2. The study was conducted according to the Declaration of Helsinki and approved by the local ethical board (ethical vote: 567/2015BO2). Sample characteristics are shown in Table 1.

**TABLE 1:** Sample characteristics (means, standard deviations and range).

	BN (*n* = 21)	BD^+^ (*n* = 20)	HC (*n* = 20)	Test statistics	*p*
Age (years)	24.4 (6.3) [19–43]	27.5 (12.2) [19–68]	25.2 (7.3) [18–53]	*F*(2, 58) = 0.34	0.714
Years of education	14.2 (2.2) [13–18]	15.1 (2.5) [13–18]	14.5 (2.4) [13–18]	*F*(2, 56) = 0.67	0.517
Non‐German citizenship	5	2	0	*χ* ^2^(2) = 5.78	0.056
BMI (kg/m^2^)	22.6 (2.4) [18.3–27.8]	24.1 (4.6) [17.0–34.8]	21.4 (1.8) [18.0–24.0]	*F*(2, 58) = 3.15	0.050
BSQ	144.8 (19.9)^a^ [105–184]	136.6 (26.2)^a^ [96–187]	53.6 (10.1)^b^ [34–66]	*F*(2, 58) = 132.3	< 0.001
EDE‐Q global score	3.8 (0.9)^a^ [1.3–5.1]	3.2 (1.2)^a^ [0.8–5.4]	0.3 (0.3)^b^ [0.0–1.0]	*F*(2, 58) = 90.1	< 0.001
EDE‐Q shape concern	4.3 (1.1)^a^ [1.6–6.0]	4.2 (1.1)^a^ [1.4–5.6]	0.6 (0.5)^b^ [0.0–2.0]	*F*(2, 58) = 115.4	< 0.001
EDE‐Q weight concern	3.9 (0.9)^a^ [1.8–5.4]	3.3 (1.5)^a^ [0.8–5.4]	0.2 (0.3)^b^ [0.0–1.2]	*F*(2, 58) = 72.95	< 0.001
EDE‐Q eating concern	2.7 (1.3)^a^ [0.4–5.6]	1.8 (1.4)^a^ [0.0–4.4]	0.0 (0.1)^b^ [0.0–0.2]	*F*(2, 58) = 31.72	< 0.001
EDE‐Q restraint	3.4 (1.0)^a^ [1.0–4.6]	2.7 (1.9)^a^ [0.0–6.0]	0.1 (0.2)^b^ [0.0–0.8]	*F*(2, 58) = 34.95	< 0.001

*Note*: Continuous variables and categorical variables were compared via one‐way ANOVA and Pearson's Chi‐squared tests, respectively. Means with different superscripts differ at the *p* < 0.001 level, measured via a two‐sided between‐subject Welch *t*‐test.

Abbreviations: BD^+^, High Body Dissatisfaction Group; BMI, body mass index; BN, Bulimia Nervosa Group; BSQ, Body Shape Questionnaire; EDE‐Q, Eating Disorder Examination‐Questionnaire; HC, Healthy Control Group.

### 2.2 Questionnaires and Interviews

The following validated instruments were used: (1) *Eating Disorder Examination* (EDE, Hilbert and Tuschen‐Caffier 2016) to establish the presence/absence of current eating disorder diagnoses (AN, BN, binge eating disorder, other specified eating and feeding disorders) via thirteen diagnostic items, which were further modified to assess lifetime presence/absence of eating disorders, (2) *Structured Clinical Interview for DSM‐IV* (SCID‐I and –II, Wittchen et al. 1997) to diagnose the presence/absence of mental disorders other than an eating disorder (at the time of the start of the study, no German version of the SCID for DSM‐5 was available), (3) *Eating Disorder Examination‐Questionnaire* (EDE‐Q, Hilbert et al. 2007) to assess severity of eating pathology, (4) *Body Shape Questionnaire* (BSQ, Pook et al. 2002) to measure appearance concerns and body dissatisfaction. For further information regarding psychometric properties see Appendix S3.

### 2.3 Stimuli and Task

**FIGURE 1:**
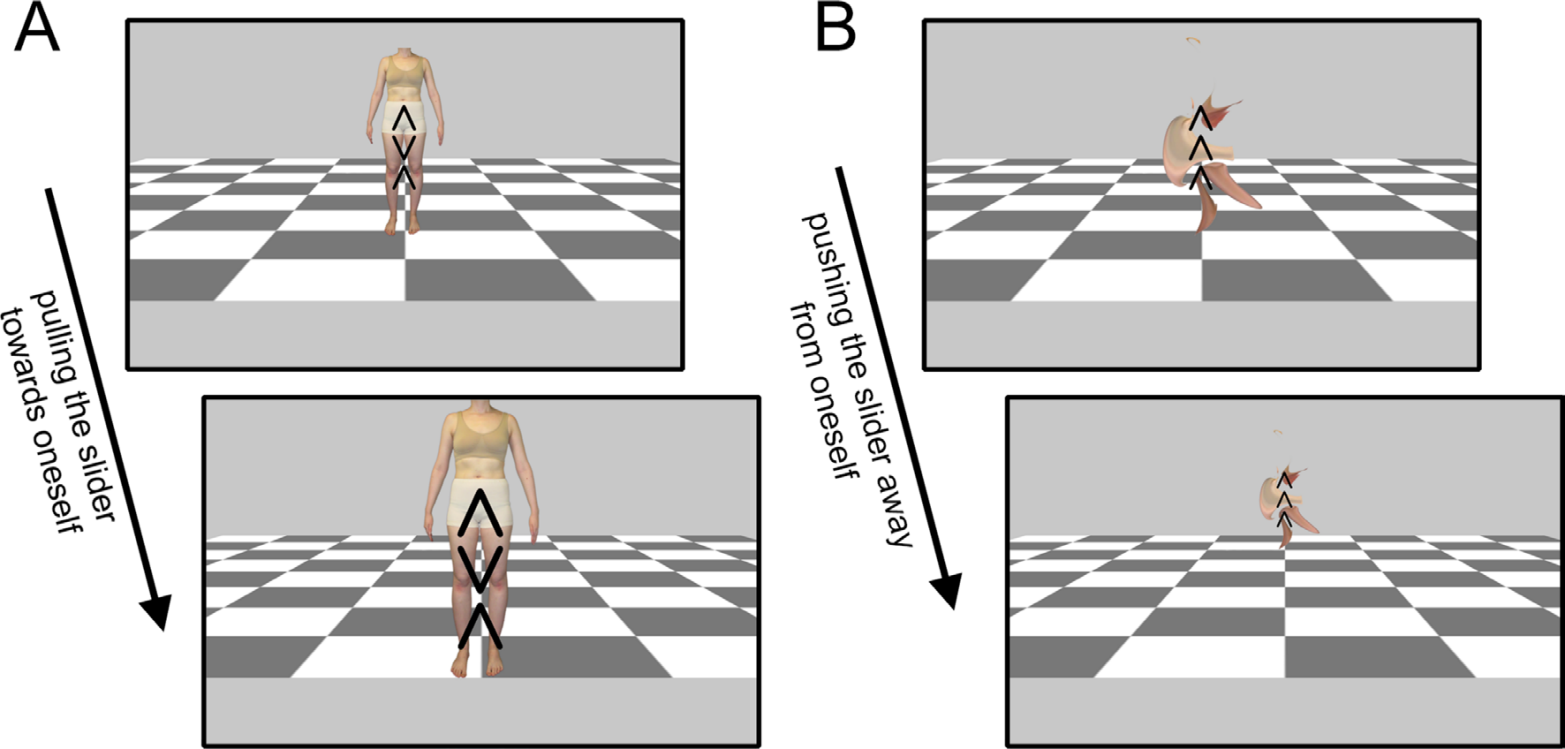
Example AAT trials. Depicted are both the initial and the end position (after the correct slider response) of the stimuli. Panel A: body image incompatible flanker pull trial; panel B: neutral image compatible flanker push trial.

Twelve pictures of each participant's own body and twelve neutral pictures were used as stimulus material for the AAT. Body pictures were taken in standardized underwear and in twelve predetermined positions. Neutral pictures were generated using diffeomorphic transformation (Press et al. 2022) of the body images, ensuring the exact same visual characteristics and a similar shape, while the body itself was not recognizable. The pictures were presented superimposed by the flanker arrows on a gray background with a checkerboard pattern (see Figure 1 for example pictures).

The task consisted of four blocks with 96 trials each. The participant initiated each trial by positioning the slider device to a central position (for a photograph of the slider device see Appendix S4) with visual feedback guiding this movement. A magnetic brake controlled by experiment software was applied during a 1.5 s interval where a fixated cross was presented. Then, the brake was released, and a picture (body or neutral) was presented, along with the flanker display. The two flanking arrows pointed in the opposite (incompatible trial) or same (compatible trial) direction as the central target arrow. Participants were instructed to either push or pull a slider with their preferred hand, depending on the direction of the central arrow. The push/pull movement of the slider controlled the stimulus display: as the participants moved the slider, the picture and flanker became smaller (were pushed) or bigger (were pulled) within the 3D scene. Participants were required to push the slider until it reached the full movement extent (approximately 18 cm). The slider device was sampled at approximately 250 Hz, calibrated to provide online distance (in cm) from the central position during a trial. The resulting time point by distance data was re‐analyzed offline (see Appendix S5 for a detailed description of the data preparation) to provide accurate movement onset and offset values. The task was programmed in MATLAB (Version R2016a) using the Psychophysics toolbox extensions (Brainard 1997; Kleiner et al. 2007; Pelli 1997).

### 2.4 Procedure

In the first laboratory session, diagnostic interviews were conducted by trained interviewers, after which the standardized body pictures were taken in a designated additional laboratory room. The second part of the experiment was completed on a different day to reduce participant burden regarding session duration, as well as to eliminate possible explicit effects induced through schema activation while being photographed in underwear. After providing informed consent, they filled in the questionnaires and completed a task unrelated to the present study. After a break, they received instructions for the AAT and completed the task (45 min). See Appendix S6 for further details.

### 2.5 Statistical Analysis

We assessed two dependent variables: time until a reaction was initiated (movement onset) and time between initiation of a response and its conclusion (movement duration). The three within‐participant independent variables were Picture Type (body vs. neutral), Movement Direction (push vs. pull) and Flanker Compatibility (compatible vs. incompatible, respectively low vs. high cognitive load) for the three groups (BN, BD^+^, HC).

Analyses were conducted in R (4.4.1 R Core Team, 2024), using the lme4 package (Bates et al. 2015) via the mixed function within the afex package (Singmann et al. 2024) for calculating linear mixed models and the emmeans package (Lenth 2024) for follow‐up analyses. In the linear mixed effects model, we implemented fixed effects of group, compatibility, picture type and response direction, and random intercepts for each participant. Statistical significance (with *α* = 0.05) was assessed by likelihood‐ratio tests.

We used linear mixed models for the analysis as they are more robust to missing data and are superior in controlling for Type I errors than alternative approaches (Gueorguieva and Krystal 2004; Yu et al. 2022). Nevertheless, due to the particular partitioning of variance in linear mixed models, no consensus currently exists on a universally accepted method for calculating standardized effect sizes for main effects and interactions (Rights and Sterba 2019). Therefore, we follow the recommendation to report unstandardized effect sizes (Pek and Flora 2018), which are represented by the estimated fixed effects (*β*).

## 3 Results

### 3.1 Movement Onset

Contrary to our main hypothesis, there was no significant three‐way interaction of Group × Picture Type × Movement Direction for movement onset, *χ* ^2^(2) = 0.98, *p* = 0.613. The estimated fixed effect for the three‐way interaction for BD group, body picture type, and pull movement direction is *β* = −1.55 ms, 95% CI [−17.94, 14.85] and for BN group, body picture type, and pull movement direction *β* = 4.31 ms, 95% CI [−11.85, 20.47]. The estimated fixed effects represent additive changes in movement onset time from the reference factor levels HC group, neutral picture type, and push movement direction.

Regarding lower level two‐way interactions, we observed a significant interaction of Picture Type × Movement Direction, *χ* ^2^(1) = 7.15, *p* = 0.007. Participants across groups showed a significant approach‐bias in their movement onsets, i.e., they initiated movements faster to pull than to push instructions for both picture types (both *z* > 3.56, *p* < 0.001), but this bias was larger for body images than neutral images (*z* = 2.68, *p* = 0.008, *β* = 6.44 ms, 95% CI [1.71, 11.16]). There furthermore was a significant Group × Movement Direction interaction, *χ* ^2^(2) = 7.51, *p* = 0.023. Again, follow‐up tests showed participants in all groups reacted significantly faster in pull than in push trials (all *z* > 3.26, *p* < 0.002); however, this effect was significantly larger in the BN than in the HC group (*z* = 2.47, *p* = 0.036, *β* = 7.20 ms, 95% CI [1.47, 12.92]), whereas other group comparisons were not significant (both *z* < 2.25, *p* > 0.06).

Regarding our exploratory analysis of cognitive load, no modulating effect of the Flanker Compatibility was found when adding it as a component in a four‐way Flanker Compatibility × Group × Picture Type × Movement Direction interaction, *χ* ^2^(2) = 0.03, *p* = 0.985. We did observe a significant Group × Compatibility interaction, *χ* ^2^(2) = 6.83, *p* = 0.033, with follow‐up tests showing that participants in all three groups reacted faster to compatible flanker trials than to incompatible trials (all *z* > 19.09, *p* < 0.002), but this compatibility effect was larger for the HC group compared to the BN group (*z* = 2.5, *p* = 0.028, *β* = 7.47 ms, 95% CI [1.75, 13.19]). In contrast, the other between‐group comparisons were not significant (all *z* < 1.74, *p* > 0.19).

**FIGURE 2:**
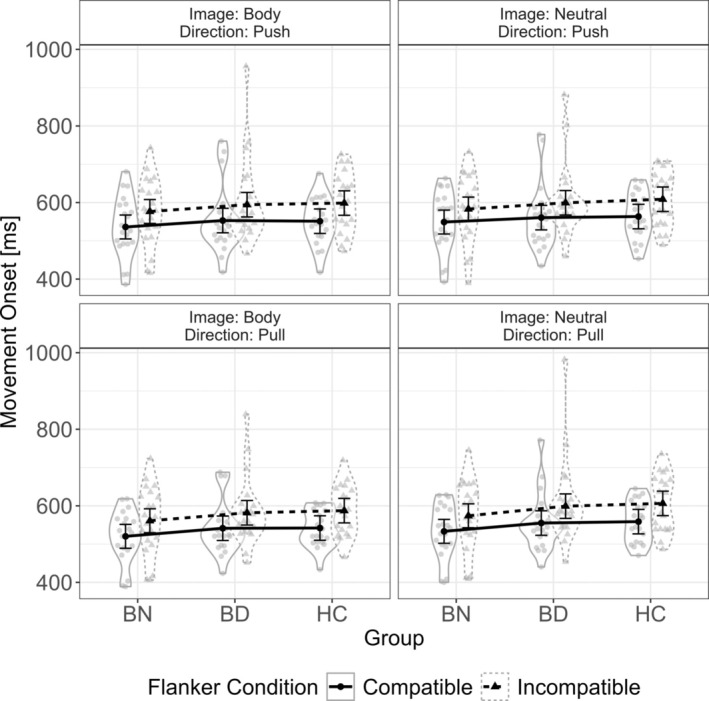
Movement onset time as a function of image type, movement direction, and flanker compatibility for each group. BD^+^, High Body Dissatisfaction Group; BN, Bulimia Nervosa Group; HC, Healthy Control Group. Mean values of movement onset by group and condition are depicted with error bars attached. Mean movement onset time for each participant in each condition and group is visualized by gray data points within the violin plot. The width of the violin depicts the probability density of the data.

Results are depicted in Figure 2.

### 3.2 Movement Duration

In contrast to our hypothesis but consistent with the results from the movement onset analysis, for movement duration there was no significant three‐way interaction of Group × Picture Type × Movement Direction, *χ* ^2^(2) = 0.95, *p* = 0.623. The estimated fixed effect for the three‐way interaction for the BD group, body picture type, and pull movement direction is *β* = −3.77 ms, 95% CI [−15.20, 7.65] and for the BN group, body picture type, and pull movement direction *β* = 1.69 ms, 95% CI [−9.57, 12.96]. The estimated fixed effects represent additive changes in movement duration from the reference factor levels HC group, neutral picture type, and push movement direction.

We observed a significant two‐way interaction of Group × Movement Direction, *χ* ^2^(2) = 24.18, *p* < 0.001. Follow‐up tests showed that across picture types, participants completed push movements significantly faster than pull movements (i.e., displaying an avoidance bias for both picture types), in the BN (*z* = 8.30, *p* < 0.001, *β* = 11.80 ms, 95% CI [9.02, 14.58]) and HC (*z* = 5.52, *p* < 0.001, *β* = 8.04 ms, 95% CI [5.18, 10.90]) groups, while no significant difference in movement duration for push and pull movements was shown in the BD^+^ group (*z* = 1.21, *p* = 0.223).

Regarding the exploratory analysis of cognitive load, no significant four‐way interaction of Flanker Compatibility × Group × Picture Type × Movement Direction was observed, *χ* ^2^(2) = 0.38, *p* = 0.828. There was a significant Compatibility × Movement Direction interaction, *χ* ^2^(1) = 4.57, *p* = 0.033. While participants generally completed movements faster during compatible than incompatible flanker trials, this effect was larger for push than pull movements (*z* = 2.14, *p* = 0.032, *β* = −3.59 ms, 95% CI [−0.30, −6.88]).

**FIGURE 3:**
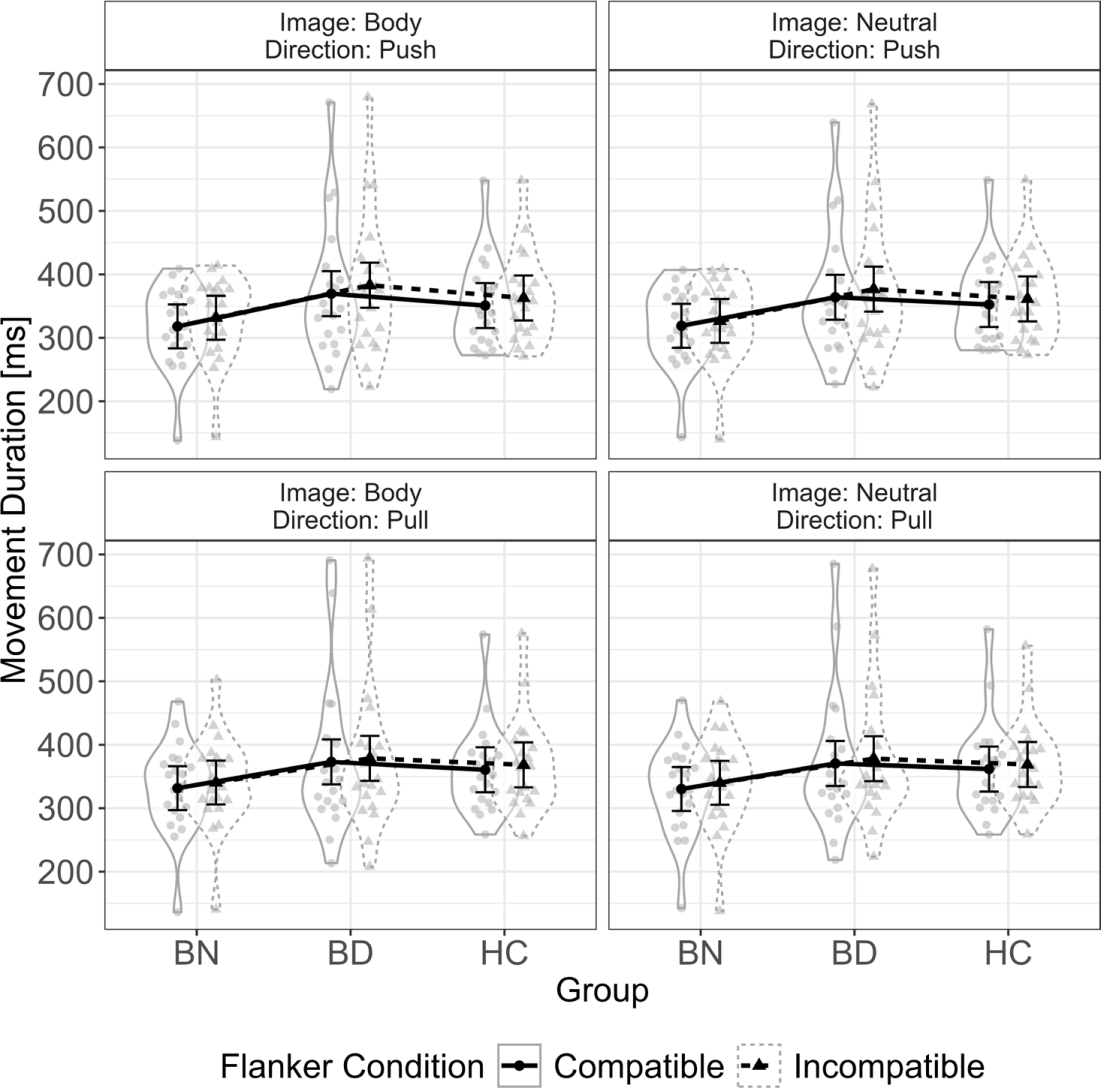
Movement duration as a function of image type, movement direction, and flanker compatibility for each group. BD^+^, High Body Dissatisfaction Group; BN, Bulimia Nervosa Group; HC, Healthy Control Group. Mean values of movement duration by group and condition are depicted with error bars attached. Mean movement duration time for each participant in each condition and group is visualized by gray data points within the violin plot. The width of the violin depicts the probability density of the data.

Results are visualized in Figure 3.

In addition, the distribution of the flanker effect shows a standard positive going slope. That is, the compatibility effect increases with slower responses (De Jong et al. 1994; Mackenzie and Dudschig 2021; Ulrich et al. 2015) across groups (visualized in Appendix S7).

## 4 Discussion

The present study assessed avoidance behavior in females with BN and females with high BD by the AAT using self‐depicting body images and perceptually matched neutral images to get a better understanding of motivational tendencies within body image disturbances. We further wanted to investigate how cognitive load affects AAT‐evoked biases as a contributing factor to prior discrepant findings. Therefore, in the present study, participants reacted to the superimposed flanker task and not to the image content.

Contrary to our hypothesis, there was no evidence of an avoidance bias of self‐depicting body images versus neutral images in the BN and the BD^+^ compared to the HC group for either movement onset or movement duration. Furthermore, cognitive load (compatible vs. incompatible flanker trials) had no effect. A standard flanker effect was, however, observed, with participants reacting slower to incompatible flanker trials, yielding evidence for the validity of the basic paradigm used.

While the approach‐avoidance research is rather consistent in the domain of phobias and addictions, with several replications of distinct approach‐avoidance biases (Field et al. 2011; Heuer et al. 2007; Klein et al. 2011), as well as promising AAT‐based trainings (Eberl et al. 2014; Kakoschke et al. 2017), results of AAT studies in the domain of body image disturbances are much more heterogeneous. Several reasons may account for this discrepancy.

For one, only a few studies have used the AAT in body image research, and this paucity is further complicated by the variety of the AAT, which ranges from the use of joysticks, manikins, and touchscreens. Comparing joystick‐and manikin‐based AAT, the latter consistently produced larger effect sizes, regardless of whether the valence of the stimulus was task‐relevant or irrelevant (Krieglmeyer and Deutsch 2010). It may therefore be more sensitive to valence‐induced activation of approach‐avoidance responses. Moving the manikin may be conceptualized as *moving* towards and away from the stimulus (i.e., walking or running towards and away from something). In contrast, the conceptualization of arm extension and flexion in the context of joystick‐based AAT is not as straightforward. When pushing away something, one must first make contact with it in order to move it. This may be counterintuitive if the behavioral purpose is avoidance. Furthermore, arm extension could also be interpreted as reaching for the stimulus, while arm flexion could imply withdrawing the hand from something. This may further confound the anticipated effects. Therefore, moving oneself away from or towards the stimulus may be the more natural and direct response, and therefore more automatic, than moving the stimulus itself.

The relevance of AAT variety is further supported by a study that examined the importance of visual whole‐body motion information in the AAT. Comparing different AAT modalities, it was shown that large and replicable effects were observed when the AAT simulated the visual information generated by whole‐body motion. In contrast, only a small effect was observed in the manikin task, and no significant effect was found when the stimulus was moved (Rougier et al. 2018). In the case of body images, it was further hypothesized that moving a self‐representing manikin towards body images could represent the desire to attain such a body shape (Dondzilo et al. 2021), which could activate further underlying motivational schemata, thus reinforcing the approach‐avoidance bias.

In the present study, we used a novel slider construction that largely mimics the joystick setup. Although this slider construction included stronger sensorimotor aspects via larger arm movements paired with visual feedback (i.e., zooming in and out of the stimulus according to the push or pull movement) compared to joystick setups, it may have reduced the sensitivity to detect an avoidance bias because it did not display whole‐body movement. Future studies should therefore compare different AAT methods, focusing on the advantages and disadvantages for studies involving (self‐depicting) body images.

Of note, with the addition of the flanker task, the content of the images was irrelevant to the push and pull motion (i.e., the avoidance or approach). While some studies have found evidence for approach–avoidance biases even when the stimulus eliciting the bias is task‐irrelevant (Brockmeyer et al. 2020; Krieglmeyer and Deutsch 2010; Rinck and Becker 2007), other studies suggest that the task relevance is critical for producing an approach–avoidance bias (Field et al. 2011; Lender et al. 2018; Meule et al. 2019; Mirabella et al. 2023; Welsch et al. 2023). At least in studies that used non‐self‐depicting body stimuli and compared thin and non‐thin bodies, effects were more often found in AATs where the stimuli were task‐relevant (Dondzilo et al. 2019; Dondzilo et al. 2021; Woud et al. 2011), whereas null effects were reported in studies where the body images were task‐irrelevant (first experiment in Brockmeyer et al. 2020; Kollei et al. 2022; Leins et al. 2018).

It is further important to mention that participants in our study may have been too focused on the flanker stimulus, thereby preventing any measurable automatic processing of the body images. In particular, the placement of the flanker arrows in the center of the stimuli may have obscured body parts that are often indexed as unattractive, thereby dampening the urge to engage in avoidance behavior. At the same time, and as depicted in Figure 1, it is important to note that, while undoubtedly dominant, the flankers still enabled a processing of the various body parts. Furthermore, several studies conducted with females with eating disorders and/or high BD have reported increased levels of distress even when participants' stomachs or lower bodies had been covered by clothing (Key et al. 2002; Trentowska et al. 2017; Tuschen‐Caffier et al. 2003). Nonetheless, future studies should present the flankers in a less dominant manner. Furthermore, these studies should include task relevance as a factor and ensure adequate stimulus processing for a maximized effect.

Notwithstanding the importance of the task‐relevance factor, a previous study (Brockmeyer et al. 2020) found an approach bias for self‐depicting thin images compared to other‐depicting thin images, even though body size and self‐reference were task‐irrelevant. This study argued that self‐reference is an important facilitator of approach‐avoidance effects induced by body dissatisfaction, as self‐reference has been shown to increase attentional focus (Tacikowski and Nowicka 2010). While the present study included self‐depicting images of the participant, it also displayed the pictures without the face to limit the visual information to the body stimulus. By contrast, Brockmeyer et al. (2020) manipulated the face of the depicted bodies (own vs. other face). Previous studies suggest that face and body stimuli are processed differently with respect to self‐recognition. Whereas for faces, visual recognition of visual features is the proposed mechanism underlying self‐recognition (Brady et al. 2005; Tong and Nakayama 1999), for bodies, individuals rely more on multisensory integration (van den Bos and Jeannerod 2002). Therefore, simply presenting the image of the participant's body may not have been sufficient to elicit the necessary level of self‐recognition.

It is noteworthy that inconsistent findings have also been reported in studies that employed a self‐depicting training version of the AAT to reduce body image‐related symptomatology (Glashouwer et al. 2020; Kollei et al. 2017). In these studies, participants were actively trained to push away thin versions of their own body or thin‐ideal body pictures and were instructed to pull realistic self‐images closer. While Glashouwer et al. (2020) found no effect of the AAT training on body‐related outcomes when compared to a placebo and a no‐training control condition, Kollei et al. (2017) found that participants in the training condition self‐reported a higher body satisfaction as well as reduced eating disorder symptoms when compared to a waitlist control group. Notably, in Glashouwer et al. (2020) full body images were utilized, while in Kollei et al. (2017), the self‐depicting images did not display the participants' faces. However, in this latter study, the AAT intervention also encompassed positive and negative written statements as stimuli, in addition to a brief counseling session and self‐depicting body pictures. Consequently, it is not possible to distinguish the impact of the AAT intervention from that of the other treatment components.

One more aspect to consider in body‐related AAT is that body‐related avoidance often co‐occurs with checking behavior (Fairburn et al. 1999; McLean and Paxton 2019; Trottier et al. 2015). In the present study and inherent to the AAT, participants could not completely avoid seeing their body as they would normally do when engaging in real‐world body avoidance behaviors (e.g., covering the body with oversized clothing or not looking in mirrors). Although body images are pushed away in the AAT, they remain visible until the end of the trial—and may even reappear in the next trial, regardless of the participant's response. Therefore, it is possible that body checking tendencies are activated because complete avoidance is not possible. This could lead to an internal competition between the avoidance and checking behavior, in turn masking the effects in the implicit measure. To account for body checking, future studies should at least include a trait measure of body checking, but also include items tackling momentary checking behaviors and their changes throughout the task. Alternatively, future studies could experimentally manipulate checking behavior, e.g., by administering the AAT in a condition with high self‐reference (e.g., in front of an open mirror) in comparison to a condition with lower self‐reference (e.g., covered mirror).

Taken together, task‐relevance, self‐reference, stimulus properties, and AAT task modalities may all contribute to the result heterogeneity across the various body‐related AAT tasks. Against this, approach/avoidance biases are much more reliable in phobia and addiction research, even when presented with similar AAT‐related challenges (Field et al. 2011; Heuer et al. 2007; Klein et al. 2011). Therefore, it is important to critically reflect on the number of null findings in body AAT research from a theoretical perspective. While avoidance behavior is a defining *diagnostic* feature of anxiety disorders and approach behavior (i.e., intensified craving and excessive consumption) is inherent to addiction disorders, avoidance behavior in the context of body image disturbances is rather an associated (but not diagnostically required) feature. Closely related, approach‐avoidance biases may not be the crucial mechanism regarding BN (and body dissatisfaction) and other factors such as emotion regulation and/or attentional biases may be of greater relevance.

As a first limitation, in our power simulation, we defined the effect of interest as a difference of 50 ms in reaction time. It is possible that the true effect is indeed smaller, which would in turn reduce the statistical power of our study design. Second, several participants noted that pushing and pulling the slider became increasingly physically demanding. This may have additionally affected the results. Third, our sample consisted of adult, mostly highly educated females only. This selective sample, while increasing internal validity, limits the generalizability of our findings. Fourth, we did not assess the duration of illness for the BN group, as well as the lifetime diagnosis of any eating disorder for the BN and the BD^+^ groups. Lastly, this study has not been preregistered. While all analyses were theory‐driven and planned prior to data inspection, drafting these steps in a preregistration would have further enhanced transparency.

Taking into account the limitations, the results of the present study found no implicit avoidance bias regarding self‐bodies in females with BN or high BD when compared to healthy controls. Future studies should investigate the relevance of body checking during AAT (e.g., by explicit manipulation), self‐reference with and without including facial cues, and the importance of task relevance to expand the understanding of the underlying mechanisms regarding self‐body avoidance.

## Author Contributions

**Johanna Xemaire:** writing – review and editing, writing – original draft, formal analysis, visualization, validation. **Ines Wolz:** writing – original draft, writing – review and editing, conceptualization, formal analysis, project administration. **Dustin Werle:** writing – review and editing, software. **Carolin Dudschig:** writing – original draft, writing – review and editing, software, visualization. **Jennifer Svaldi:** conceptualization, supervision, project administration, writing – review and editing.

## Conflicts of Interest

The authors declare no conflicts of interest.

## Supporting information


**Data S1:** eat24523‐sup‐0001‐supinfo.docx.

## Data Availability

Code and anonymized data are available via request to the authors.
