# Supplementary material for: Measuring Implicit Approach‐Avoidance Tendencies Using Self‐Depicting Body Pictures in Female Adults With Bulimia Nervosa, High Body Dissatisfaction and Healthy Controls
Source: Int J Eat Disord. 2025 Aug 9;58(11):2126–37. doi: 10.1002/eat.24523 (PMC12605784; doi:10.1002/eat.24523)
Supplement: Supplementary file 1 — Data S1: eat24523‐sup‐0001‐supinfo.docx. [file EAT-58-2126-s001.docx]

**Supplementary Appendix**

**Appendix 1 – Power Simulation**

To specify the sample size, we created a computer simulation (Wickelmaier, 2022) through which we estimated the power of our statistical analysis. In this simulation we predefined the estimated fixed effects and the variances of the random effects in the underlying data-generating linear mixed effect model (specified in 2.4) based on AAT literature, as well as data from unpublished pilot studies. Representing the hypothesized avoidance bias for self-body images, we determined a 50 ms difference between push and pull responses for self-body images in the BN and the BD^+^ group as of interest. Simulating data using this underlying effect and α = .05 for the statistical test of the Group × Picture Type × Motion Direction interaction, a total sample size of N = 60 yielded a power of 1-β = .80. The simulation was done using R (4.4.1 R Core Team, 2024) and the code is available upon request.

**Appendix 2 – Sample Recruitment**

Participants were recruited through mailing lists and advertisements at the local university, flyers distributed in the community (e.g. pharmacies) and the affiliated outpatient clinic. To be eligible, participants had to be female,18 years or older and possess adequate proficiency in German. Exclusion criteria were a current psychotic, bipolar, post-traumatic stress or substance-related or addictive disorder. Participants received either course credit or monetary reimbursement for their participation.

**Appendix 3 – Questionnaires and Interviews**

The EDE is a structured interview for the classification and assessment of eating disorder psychopathology. Thirteen items can be used for the diagnosis of eating disorders according to DSM-5 criteria (American Psychiatric Association, 2013), 21 more items relate to four subscales (eating concern, weight concern, shape concern and restraint) regarding the assessment of specific eating disorder psychopathology in the last 28 days.

The EDE-Q assesses the presence of eating disorder symptoms in the last 28 days on four subscales: eating concern, weight concern, shape concern and restraint. Internal consistency for the four subscales in our sample were good to excellent with Cronbach’s *α* ranging from .89 (Eating Concern scale) to .95 (Shape Concern scale).

The BSQ contains 34 items which are answered on six-point Likert scales (1 = “never”; 6 = “always”) and added to a total score ranging from 34 to 204, where higher values relate to higher body dissatisfaction. In a female community sample (Cooper et al., 1987), a mean value of 81.5 (SD = 28.4) was found, while patients with BN showed a BSQ mean of 136.9 (SD = 22.5). Internal consistency in our sample was excellent with *α* = .98.

**Appendix 4 – Slider Device**

**Figure 1**
*Picture of the slider device with the slider at the initial starting position.*


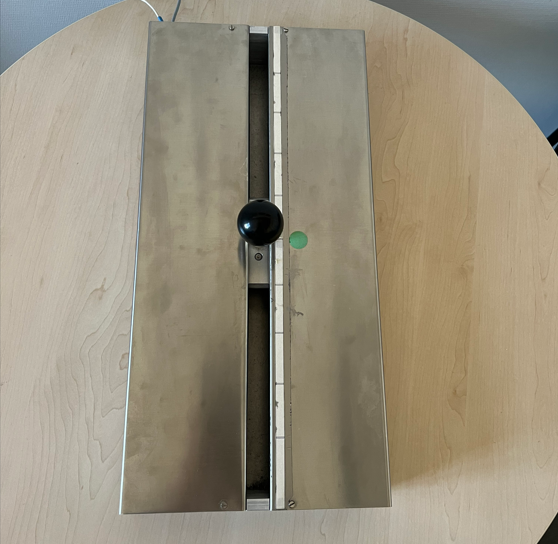


**Appendix 5 – Data Preparation**

The slider data was baselined to a 200 ms interval starting before stimulus onset. Movement onset was set to 0.5 cm, whilst movement offset was set at 15 cm (see Figure 1 for an example trial). The device was calibrated such that the distance from the initial central position was returned online and was used to update the stimulus display. Offline, the movement onset and movement offset times were calculated according to pre-defined criterion of 0.5 cm (onset) and 15 cm (offset) from the central (0 cm) position. The interval between movement onset and movement offset was taken as the movement duration. Movement onsets less than 150 ms and greater than 1200 ms and movement offsets greater than 2500 ms were considered outliers (<1 % of trials).

**Figure 2**
*Example Trial Data Recorded from the Slider Device.*


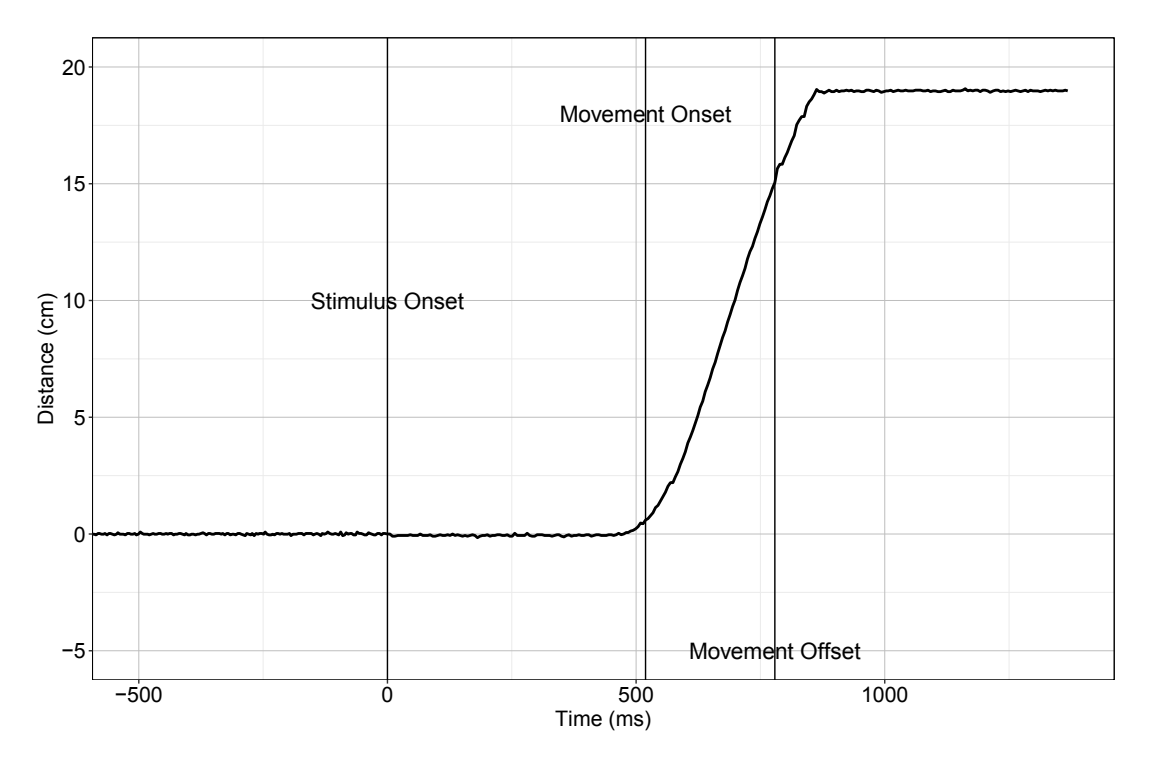


**Appendix 6 – Procedure**

Participants were first screened for body dissatisfaction using an online assessment of the BSQ. Scores between 67 and 95 led to study exclusion. For those in the pre-specified BSQ range, a telephone screening was used to assess the presence of any additional exclusion criteria (e.g. insufficient German skills) and if none were present at this stage, participants were invited to a diagnostic interview, where trained interviewers administered the EDE (diagnostic items including assessment of weight and height), SCID-I and -II in a face-to-face session. Following this, body pictures were taken in a room with standardized light conditions and in a set of standardized underwear (sports bra and underpants in beige colour). Pictures were taken in twelve standardized perspectives (head always omitted): frontal with the arms at the side of the body, then turning to the right in each position of the clock always with the arms positioned so that they do not cover the torso. Following this, participants completed the questionnaires presented on the online-platform Unipark (https://www.unipark.com). In a second laboratory session, participants took part in two tasks with a break in between, where the second was the AAT reported here (the other combined EEG/Eye Tracking task will be reported elsewhere). Participants were seated in front of a computer screen. The handling of the slider was explained, instructions and training of the task were automatized, but participants could ask any questions before starting the task, which took approximately 45 minutes. Experimental procedure is visually summarized in Figure 2.

**Figure 3**
*Visualization of the study procedure.*


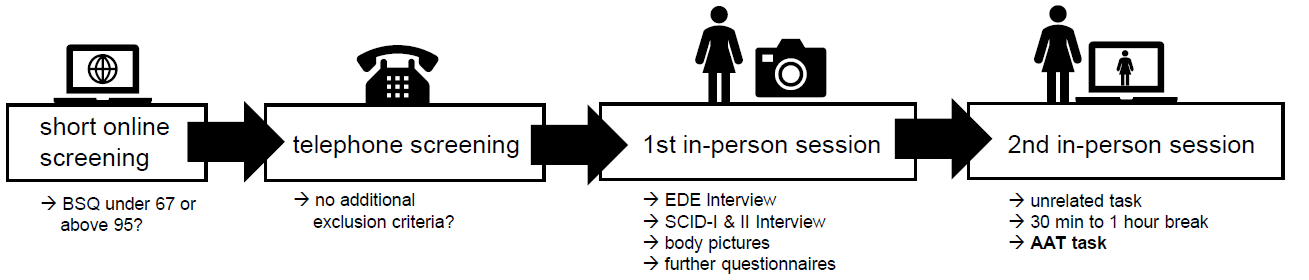


**Appendix 7 – Visualization Compatibility Effect**

**Figure 4***Compatibility Effect (Incompatible RT – Compatible RT) as a Function of Reaction Time.*

**References**

American Psychiatric Association. (2013). *Diagnostic and Statistical Manual of Mental Disorders, Fifth Edition*. American Psychiatric Association.

Cooper, P. J., Taylor, M. J., Cooper, Z., & Fairbum, C. G. (1987). The development and validation of the body shape questionnaire. *International Journal of Eating Disorders*, *6*(4), 485–494. https://doi.org/10.1002/1098-108X(198707)6:4<485::AID-EAT2260060405>3.0.CO;2-O

R Core Team (2024). R: A Language and Environment for Statistical Computing. *R Foundation for Statistical Computing*, Vienna, Austria. https://www.R-project.org

Wickelmaier, F. (2022). Simulating the power of statistical tests: A collection of R examples. *ArXiv*. https://doi.org/10.48550/arXiv.2110.09836
